# Successful Tricuspid Valve Replacement in a Patient with Severe Pulmonary Arterial Hypertension and Preserved Right Ventricular Systolic Function

**DOI:** 10.1155/2009/108295

**Published:** 2009-06-16

**Authors:** Jamil A. Aboulhosn, Ronald J. Oudiz, Amish S. Dave, Abbas Ardehali, David J. Ross

**Affiliations:** ^1^Division of Cardiology, David Geffen School of Medicine at UCLA, Los Angeles, CA 90095, USA; ^2^Los Angeles Biomedical Research Institute at Harbor, UCLA Medical Center, Los Angeles, CA 90502, USA; ^3^Division of Cardiothoracic Surgery, David Geffen School of Medicine at UCLA, Los Angeles, CA 90095, USA; ^4^Division of Pulmonary and Critical Care Medicine, David Geffen School of Medicine at UCLA, Los Angeles, CA 90095, USA

## Abstract

A 56-year-old patient with severe pulmonary hypertension developed severe tricuspid regurgitation, right-sided heart failure, and congestive hepatopathy. She was transferred for possible lung transplant and/or tricuspid valve surgery. Clinical and echocardiographic assessment provided confidence that acute tricuspid valve failure was responsible for the decompensation and that tricuspid valve replacement despite pulmonary hypertension could be performed.

## 1. Case Presentation

**Figure 1:**
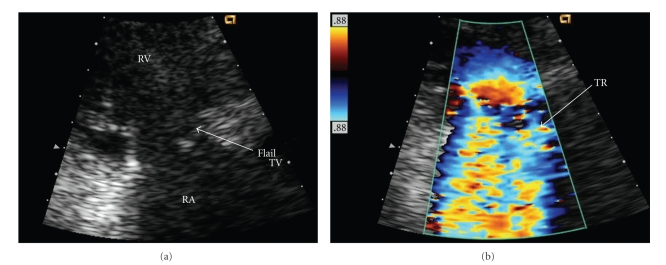
(a) Transthoracic 2D echo parasternal long-axis systolic view of a flail anterior tricuspid valve leaflet (TV). Right atrium (RA) and right ventricle (RV) are labeled. The tricuspid valve annulus is not dilated, measuring 3 cm in maximum diameter. (b) Color flow Doppler demonstrating severe tricuspid regurgitation (TR).

**Figure 2:**
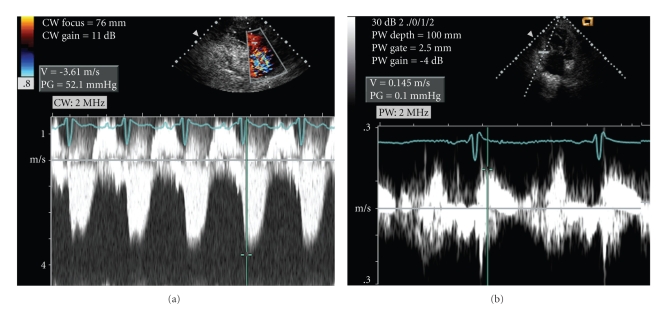
(a) Continuous wave Doppler profile of tricuspid regurgitation in the parasternal long-axis view. The tricuspid valve regurgitant velocity is 3.61 m/s. Using the modified Bernoulli equation (Δ*P* = 4*V* ^2^) and assuming a right atrial pressure of 15 mmHg, the estimated pulmonary artery systolic pressure is 67 mmHg. (b) Tissue Doppler profile of the tricuspid valve annulus demonstrates a systolic velocity of 14.5 cm/sec consistent with preserved right ventricular function [1, 2].

**Figure 3:**
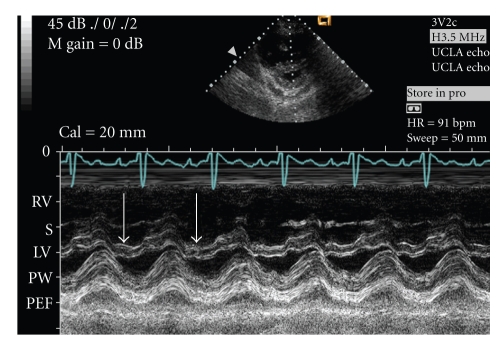
M-mode echo, parasternal short-axis, demonstrating leftward displacement of the septum during diastole (white arrows), consistent with right ventricular volume overload. Right ventricle (RV), septum (S), left ventricle (LV), posterior wall (PW), and pericardial effusion (PEF).

A-56-year old Hispanic female with idiopathic pulmonary arterial hypertension (PAH) for 13 years was stable (WHO class II) on subcutaneous treprostinil for 8 years. In 1999, diagnostic right heart catheterization had shown elevated right atrial (mean 20 mmHg) and pulmonary artery (PA) pressure (145/42 mmHg) with normal pulmonary capillary wedge pressure (10 mmHg). The patient began complaining of fatigue, lethargy, and dyspnea on minimal exertion in January 2007 and was admitted in February for syncope, anorexia, abdominal pain, jaundice, and decreasing urine output. An echocardiogram revealed right ventricular hypertrophy with severe tricuspid regurgitation (TR) and preserved right ventricular (RV) systolic function. In May 2007 she was admitted to an outside hospital for persistent symptoms and resistant volume overload. Physical examination demonstrated jaundice, ascites, and right heart failure. Her jugular venous pressure was 18 cm H_2_O. A systolic RV heave was present. There was a 3/6 holosystolic murmur at the left sternal border, a loud pulmonary component of the second heart sound and an RV S4 gallop. The patient was moderately hypotensive (systolic blood pressure of 80 mmHg), and room air oxygen saturation was 93%. An echocardiogram demonstrated severe TR with a flail anterior leaflet (Figure 1), which was not previously noted. Estimated PA pressure had fallen to 2/3 of systemic (Figure 2(a)). M-mode echocardiography demonstrated diastolic leftward septal motion consistent with RV volume overload (Figure 3). RV systolic function was unchanged, and there was no TV annular dilation.

Retrospective review of hepatic function aroused suspicion for acute decompensated RV failure with congestive hepatopathy occurring several months earlier, possibly related to the flail tricuspid leaflet. Upon review of an echocardiogram performed three months prior, the flail tricuspid leaflet was indeed found to be present. Because of the temporal relationship between the severe TR and worsening symptoms as well as RV failure and hepatic dysfunction despite preserved RV systolic function, it was felt that tricuspid valve (TV) failure alone could explain the patient's symptoms and that TV repair or replacement might benefit the patient. 

The patient was continued on subcutaneous treprostinil (46 ng/kg/min) and treated with diuretics and digoxin then transferred to our institution for evaluation for possible lung transplant and/or TV surgery. Repeat right heart catheterization demonstrated elevated right atrial pressures (*V* = 35 mmHg, mean = 21 mmHg), moderately elevated PA pressure (64/29 mmHg), pulmonary capillary wedge pressure at upper limits of normal (mean 16 mmHg), decreased cardiac index by thermodilution (1.7 L/min/m^2^), and elevated pulmonary vascular resistance (7.1 Wood Units). Chest CT demonstrated marked dilation of the main and proximal branch pulmonary arteries with mural calcification and a moderate pericardial effusion. Ventilation/perfusion scan showed low probability for thromboembolism. Laboratories showed platelet count of 58000/*μ*L, total bilirubin of 3.3 mg/dL, serum creatinine of 1.2 mg/dL, and albumin of 3.1 g/dL. Repeat echocardiogram with agitated saline demonstrated excellent biventricular systolic function, absence of tricuspid annular dilation, and late (>5 cycles) right-to-left shunting suggesting a pulmonary right-to-left shunt. The patient received empiric steroids for possible idiopathic thrombocytopenic purpura after other etiologies were excluded, with improvement of her platelet counts. The patient also developed recurrent atrial flutter. 

Because of her hyperbilirubinemia and thrombocytopenia, she was felt to be a poor candidate for lung transplantation. It was concluded that without TV surgery, the patient's long-term outcome would be poor and that surgery was indicated. The recommendation was buttressed by the presence of normal RV systolic function with normal TV tissue Doppler systolic annular velocity (Figure 2(b)) and the abrupt clinical deterioration suggesting sudden disruption of valve integrity leading to poorly tolerated severe TR. The patient was presented with the option of high-risk surgical intervention and consented to TV surgery. 

Two weeks later the patient underwent TV replacement. Treprostinil was held immediately prior to surgery. Intraoperative inspection of the TV revealed ruptured chordae with a flail, prolapsing anterior leaflet, and normal annular size. A patent foramen ovale was not present. Valve repair was not performed due to friability of the subvalvular apparatus and concern regarding longevity of repair given her pulmonary hypertension. A stented 27 mm Magna (Edwards Life Sciences) bioprosthesis was selected for TV replacement. Right atrial MAZE cryoablation and ligation of the left atrial appendage were also performed. PA systolic pressure increased to 90 mmHg postvalve replacement (preintervention baseline 65 mmHg). The patient was successfully weaned from cardiopulmonary bypass on intravenous epinephrine, dopamine, milrinone, inhaled nitric oxide (40 parts per million), and intravenous prostacyclin (20 ng/kg/min). Total cardiopulmonary bypass time was 106 minutes, and aortic cross-clamp time was 81 minutes.

Catheterization on postoperative day 1 demonstrated PA pressure of 90/32 mmHg and improved cardiac index of 2.7 L/min/m^2^ and a pulmonary vascular resistance of 6.7 Wood units. Repeat echo demonstrated decreased RV size, normal systolic function, and mild high pressure TR. She was extubated on postoperative day 4 and discharged from the intensive care unit 10 days postoperatively. She was transitioned from intravenous prostacyclin to subcutaneous treprostinil (20 ng/kg/min) and oral sildenafil (20 mg twice daily). She was discharged on postoperative day 15 on the above medications as well as digoxin, prednisone, spironolactone, bosentan, and furosemide. She was seen in clinic three months postoperatively and had returned to her baseline functional status (WHO class II). The jaundice, ascites, hypoalbuminemia, RV failure, and liver abnormalities had resolved.

## 2. Discussion

There is a paucity of data on the incidence, prevalence, and prognosis of severe TR in patients with PAH not due to elevated left heart filling pressures. It has long been thought that TV surgery in patients with severe pulmonary hypertension without mitral valve disease carried an unacceptably high risk of mortality. This patient's presentation is not unique; although uncommon, tricuspid chordal rupture can occur in patients with PAH. However, the use of multiple clinical and imaging modalities to determine that TV surgery *without* concomitant lung transplantation could be successfully performed is novel. 

The relatively sudden deterioration after a long period of clinical stability was suspicious for an abrupt event leading to right heart failure and hepatic dysfunction. Chronic TR secondary to RV enlargement and systolic failure is more likely to result in gradual functional decline. Absence of RV systolic dysfunction or tricuspid annular dilation and presence of a flail leaflet all pointed to chordal rupture as the index event leading to clinical deterioration. In patients with implantable pacemakers and cardioverter-defibrillators, disruption of the tricuspid valve apparatus by the device lead must also be considered [3].

Echocardiography with Doppler is a readily available, cost-effective, and accurate tool for the anatomic, hemodynamic, and functional assessment of cardiopulmonary status in patients with pulmonary hypertension [1, 2, 4, 5]. At centers that do not employ tissue Doppler, tricuspid annular plane systolic excursion (TAPSE) may be used to evaluate right ventricular function [6]. Computed tomography and magnetic resonance imaging can also provide an excellent assessment of right ventricular size and function [7, 8]. 

Pulmonary vasodilator therapy in PAH is associated with improved quality of life and functional performance [9]. This patient was managed with subcutaneous and intravenous prostacyclin prior to, during, and following surgery. Both phosphodiesterase inhibitions with sildenafil and endothelin antagonism with bosentan were utilized in this patient. Perioperative mortality and morbidity risk is not low, and difficult intra- and postoperative courses are common in patients with advanced PAH. 

In conclusion, this report describes successful TV replacement in a patient with severe PAH unrelated to mitral valve disease presenting with severe RV failure and hepatic dysfunction. Clinical and echocardiographic assessments suggested that increased TR severity was secondary to abrupt chordal rupture. RV systolic function and tricuspid annular diameter were preserved, supporting the hypothesis that TV competence would be well tolerated. Importantly, in patients with significantly impaired RV function, tricuspid valve surgery carries an extremely high risk of mortality and is generally contraindicated. Valve replacement was chosen over valve repair given the absence of annular dilation, the friable nature of the subvalvular apparatus, and the concern regarding long term durability of valve repair given her pulmonary hypertension. A bioprosthetic valve was selected over a more durable mechanical prosthesis because of the patient's thrombocytopenia and the wish to avoid chronic anticoagulation. The patient has done remarkably well in the twelve months following surgery. However, the long-term durability of a bioprosthetic valve in the tricuspid position in a patient with severe pulmonary hypertension is unclear. Finally, the availability of oral, subcutaneous, inhaled, and intravenous pulmonary arterial vasodilators in the current era, in addition to improved multimodality diagnostic accuracy necessitates reconsideration of a long discarded surgical option.
